# Exploring the Potential Rescue Effects of SorCS1, a Novel Amyloid Beta Competitor, on Synaptic and Cognitive Defects in an Alzheimer’s Disease Model Mouse

**DOI:** 10.1002/alz.084750

**Published:** 2025-01-03

**Authors:** Nayoung Yi

**Affiliations:** ^1^ Institut de recherches cliniques de Montréal (IRCM), Montréal, QC Canada

## Abstract

**Background:**

Soluble Aβ oligomers (AβOs) induce synapse dysfunction, leading to cognitive impairment and memory deficits in Alzheimer’s disease (AD). Our laboratory and several research groups characterized neurexin family members' physiological roles, pivotal synaptic adhesion molecules for development, plasticity, and maintenance. Beyond their normal functions, we found neurexins binding to AβOs causes AβO‐induced neurexin dysregulation. Another group’s findings highlighted SorCS1’s role, regulating neurexin1β isoform (NRX1β) trafficking and surface expression. Our recent in vitro study showed SorCS1 competitively binds NRXβ, countering AβOs' effect, rescuing AβO‐induced NRXβ dysregulation and synaptic toxicity. NRXβ’s pivotal synaptic and brain function roles suggest SorCS1 mitigates AβO‐induced synaptic dysfunction and AD‐related deficits. We hypothesize SorCS1 stabilizes synaptic NRXβ by competitively inhibiting AβO‐NRXβ interaction.

**Method:**

To explore SorCS1’s in vivo roles, we created a forebrain‐specific inducible SorCS1 transgenic mouse line, breeding it with 5xFAD, an AD mouse model overproducing AβOs. To achieve temporal and forebrain‐specific expression of SorCS1, SorCS1KI mice were crossed with CaMKIIa‐CreERT2 mice, allowing Cre recombinase expression upon tamoxifen treatment specifically in forebrain excitatory neurons. Through these crossings, we established four genetic groups: 1) non‐AD normal control(Male = 14, Female = 14, Total n = 28), 2) non‐AD with SorCS1 overexpression (OE) (Male = 12, Female = 15, Total n = 27), 3) AD model (Male = 11, Female = 12, Total = 23), 4) AD with SorCS1 OE rescue model (Male = 13, Female = 16, Total = 29). Structural, biochemical analyses such as immunoblot and immunohistochemistry, and behavioral tests (Morris water maze, Open field, Y‐maze) were conducted to determine SorCS1’s impact on synaptic impairment and memory deficits in AD mice.

**Result:**

In mice overexpressing SorCS1 in the AD model (5xFAD), no significant differences were observed in long‐term spatial learning memory (assessed using the Morris water maze), locomotor activity, or anxiety levels (evaluated in the open field test) compared to the AD control group. However, SorCS1 overexpression notably improved weakened short‐term working memory (assessed via the y‐maze) in the AD mice. Furthermore, SorCS1 overexpression partially restored the expression levels of synaptic proteins, such as PSD95, GluA1, and β‐NRX in the cortex, as well as PSD95 and α‐NRX in the hippocampus within the crude synaptosome.

**Conclusion:**

In vivo, the overexpression of SorCS1 rescues impaired levels of synaptic proteins and improves deficits in short‐term working memory.

